# Standard Colonic Lavage Alters the Natural State of Mucosal-Associated Microbiota in the Human Colon

**DOI:** 10.1371/journal.pone.0032545

**Published:** 2012-02-28

**Authors:** Laura Harrell, Yunwei Wang, Dionysios Antonopoulos, Vincent Young, Lev Lichtenstein, Yong Huang, Stephen Hanauer, Eugene Chang

**Affiliations:** 1 Section of Gastroenterology, Department of Medicine, University of Chicago Medical Center, Chicago, Illinois, United States of America; 2 Division of Infectious Diseases, Department of Medicine, University of Michigan, Ann Arbor, Michigan, United States of America; 3 Gastroenterology Institute, Rabin Medical Center, Petach Tiqwa, Israel; National Cancer Institute, United States of America

## Abstract

**Background & Aims:**

Past studies of the human intestinal microbiota are potentially confounded by the common practice of using bowel-cleansing preparations. We examined if colonic lavage changes the natural state of enteric mucosal-adherent microbes in healthy human subjects.

**Methods:**

Twelve healthy individuals were divided into three groups; experimental group, control group one, and control group two. Subjects in the experimental group underwent an un-prepped flexible sigmoidoscopy with biopsies. Within two weeks, subjects were given a standard polyethylene glycol-based bowel cleansing preparation followed by a second flexible sigmoidoscopy. Subjects in control group one underwent two un-prepped flexible sigmoidoscopies within one week. Subjects in the second control group underwent an un-prepped flexible sigmoidoscopy followed by a second flexible sigmoidoscopy after a 24-hour clear liquid diet within one week. The mucosa-associated microbial communities from the two procedures in each subject were compared using 16S rRNA gene based terminal restriction fragment length polymorphism (T-RFLP), and library cloning and sequencing.

**Results:**

Clone library sequencing analysis showed that there were changes in the composition of the mucosa-associated microbiota in subjects after colonic lavage. These changes were not observed in our control groups. Standard bowel preparation altered the diversity of mucosa-associated microbiota. Taxonomic classification did not reveal significant changes at the phylum level, but there were differences observed at the genus level.

**Conclusion:**

Standard bowel cleansing preparation altered the mucosal-adherent microbiota in all of our subjects, although the degree of change was variable. These findings underscore the importance of considering the confounding effects of bowel preparation when designing experiments exploring the gut microbiota.

## Introduction

The ability to study the enteric microbiota in health and disease has rapidly evolved, largely through the development of non-cultivation based molecular approaches that provide information on the composition and structure of complex microbial communities. In this regard, numerous studies have surveyed the colonic microbiota to determine its relationship to the host and how perturbations of it affect or are affected by diseases such as antibiotic associated colitis, inflammatory bowel disease, pouchitis, and obesity [1]–[5]. While these studies have provided a wealth of information, almost all studies of the mucosal-associated gut microbiota have sampled the colonic microbiota of the cleansed (purged) bowel following laxation, which is routinely used to prepare the colon for colonoscopic examination. Few of these studies have taken into consideration the potential confounding effect of bowel cleansing preparation on the gut microbiota that could bias results and provide misleading or artifactual information regarding the natural state of the colonic microbiota.

Mai and colleagues [6] were the first to address the effect of bowel preparation and colonoscopy on the intestinal microbiota. These investigators examined changes in the fecal microbiota in five individuals undergoing screening colonoscopy. In three of the five subjects, the microbial profiles by denaturing gradient gel electophoresis (DGGE) were different in stool samples collected after colonoscopy compared to stool collected prior to colonoscopy. This observation suggested that bowel preparations have a significant effect on the luminal (fecal) gut bacteria.

It is now recognized that the gut microbiota is a unique ecosystem consisting of numerous microbial populations working together to carry out important physiological functions. Microbial species present in the colon lumen and adherent to the colonic mucosa are most often considered for studies of the human gut microbiota [7]. The luminal microbiota fluctuate with changes in diet and luminal content, whereas the mucosa-associated microbiota is believed to be relatively stable in individuals over the course of a lifetime [8]. Stability is achieved in part through the ability of these microbes to attach to the mucosa and establish a niche through formation of biofilms and creation of selection pressures that prevent expansion of other microbial communities. From their intimate and stable association with the host, mucosa-associated microbes are likely to contribute important influences on host physiology in health and the development of disease [9]. Therefore, proper sampling of mucosal-associated microbial communities, in their natural state, is essential for better understanding of the enteric microbiota and host-microbial relationships in health and disease. For this study, we tested the hypothesis that standard colonic lavage affects the natural state of mucosal-associated microbes in the human colon.

## Materials and Methods

### Human subjects and ethics statement

Twelve healthy individuals between the ages of 25–48 years were recruited at the University of Chicago Medical Center for this study. Written consent was obtained from all subjects prior to sample collection. Subjects were excluded if they had received antibiotics in the 6 months prior to the study. The Institutional Review Board of the University of Chicago Medical Center approved this study protocol (IRB#: 15006A).

**Figure 1:**
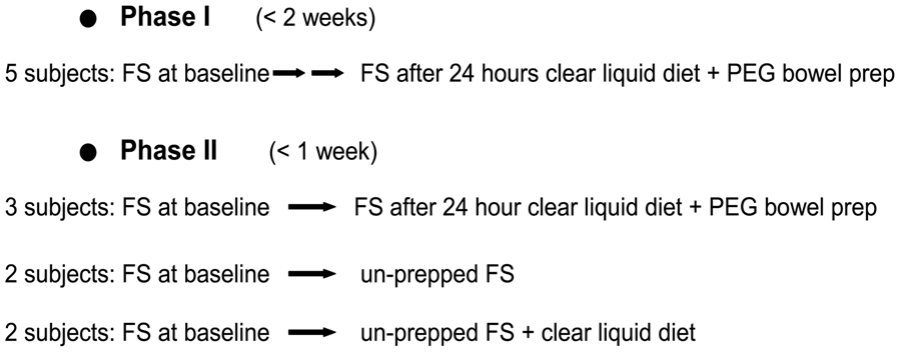
Summary of study groups.

The study design is summarized in Figure 1. In the initial phase of the study, five subjects underwent an un-prepped flexible sigmoidoscopy with mucosal biopsies (pre-prep). Biopsies were taken 20 cm from the anal verge. Within two weeks of the first flexible sigmoidoscopy, subjects were given a 24-hour clear liquid diet and a standard polyethylene glycol-based bowel cleansing preparation (Golytely®). A second flexible sigmoidoscopy with mucosal biopsies taken 20 cm from the anal verge was performed following the purge (post-prep). All biopsy samples were snap frozen at the time of collection.

In order to better understand changes in the microbiota observed during the first phase of our study, we designed a second phase. The second phase was designed to control for external factors (such as time and diet) which might alter the microbiota. In the second phase, seven healthy individuals were randomly assigned into three groups. All subjects underwent an un-prepped flexible sigmoidoscopy with mucosal biopsies (pre-prep). In order to minimize any potential variation of enteric microbial communities over time, a second flexible sigmoidoscopy was performed in all subjects within 1 week. In the first sub-group, three subjects were given a 24-hour clear liquid diet and a standard polyethylene glycol-based bowel cleansing preparation prior to the second sigmoidoscopy (post-prep). In the second sub-group, two subjects underwent a second un-prepped flexible sigmoidoscopy and were instructed to make no changes to their diet prior to the second sigmoidoscopy. In the third group another two subjects underwent a second un-prepped flexible sigmoidoscopy and were instructed to follow a clear liquid diet for 24 hours prior to the second sigmoidoscopy. All biopsies were obtained 20 cm from the anal verge.

### DNA extraction and PCR

Mucosal biopsy samples were homogenized in 1 ml extraction buffer [50 mM Tris (pH 7.4), 100 mM EDTA (pH 8.0), 400 mM NaCl, 0.5% SDS] containing 20 ul proteinase K (20 mg/ml). A slurry of 500 ul of 0.1-mm-diameter zirconia/silica beads (BioSpec Products, Bartlesville, OK) were added to the extraction tubes. Tubes were placed on a Mini-Beadbeater-8 cell disrupter (BioSpec Products) for 5 minutes lysing the microbial cells. After overnight incubation at 55°C, extraction with phenol: chloroform: isoamyl alcohol, and precipitation with ethanol were performed. Isolated DNA was dissolved in TE buffer and stored at −80°C [10], [11].

16S rRNA gene sequences were amplified from DNA templates using broad-range primers 8F (5′-AGAGTTTGATCCTGGCTCAG-3′) labeled with 6′ carboxyfluorescein (6-FAM) and 1492R (5′-GGTTACCTTGTTACGACTT-3′) for the bacterial domain [11]. PCR products were verified by electrophoresis of aliquots of PCR mixtures (8 ul) in 1.0% agarose and purified by precipitation. Aliquots of purified PCR products were digested by using *Msp*I, *Rsa*I, or *Hha*I (New England Biolabs Inc.) and subsequently subjected to capillary electrophoresis using the Applied Biosystems DNA sequencer 3130.

### Terminal restriction fragment length polymorphism (T-RFLP)

Terminal restriction fragment length polymorphism (T-RFLP) profiles were constructed for samples taken from the 5 patients enrolled in the initial phase of the study. Restriction-digestion fragment presence and abundance was determined using GeneMapper software (Applied Biosystems). Raw electropherograms were analyzed for artifacts, such as electrical anomalies, optical cross-talk between the capillaries, baseline drift, fluorescence of non-FAM-labeled contaminants, and distortions of the sizing ladder. Terminal restriction fragment (TRF) data generated by GeneMapper were filtered and binned by the method developed by Abdo et al [12]. Based on the normalized T-RFLP profile, the number and height of peaks were treated as number and abundance of bacterial phylotypes represented in samples as described previously [10]. The normalized T-RFLP profiles were used to calculate Shannon diversity indices and pairwise Bray-Curtis distances using EstimateS in order to examine the relationship between communities [13].

### 16S rRNA gene library cloning and sequencing

Library cloning and sequencing were performed on all samples obtained from the 7 subjects enrolled in the second phase of the study. Unlabeled PCR primers 8F and 1492R were used to amplify 16S rRNA gene sequences from the samples using the same protocol followed for T-RFLP analysis. PCR products were purified with the QIAquick gel extraction kit (Qiagen, Valencia, CA) and cloned into pCR-2.1-TOPO® using the TOPO-TA Cloning Kit (Invitrogen, Carlsbad, CA) according to the manufacturer's instructions. From each library, 288 colonies were picked randomly and the inserts sequenced using 8F. DNA sequencing was performed at the University of Chicago's Cancer Research Center DNA Sequencing Facility using the Applied Biosystems 3730XL DNA Analyzer.

### Sequence alignment and phylogenetic analysis

The 16S rRNA gene sequences were analyzed as previously described [10]. Raw sequence data were processed using the RDP pipeline server at the Ribosomal Database Project II (RDP-II) website (http://rdp.cme.msu.edu/pipeline) by base-calling, quality-trimming and alignment. Raw sequence data were processed and trimmed according to quality scores through an automated workflow (RDP Pipeline Tool via myRDP) available from the Ribosomal Database Project (RDP) II website (http://rdp.cme.msu.edu/) [14]. Potential chimeric sequences were identified and excluded using the SimRank 2.7 package available through the RDP and Pintail. The RDP Classifier (available via RDP-II) was used to assign 16S rRNA sequences to the taxonomical hierarchy at different levels. The program DOTUR, utilizing the furthest neighbor algorithm, was used to group sequences into operational taxonomical units (OTUs) and perform a variety of diversity analyses. OTUs were defined in this study as sequences with greater than 97% similarity. The Chao1 richness estimator adapted from mark-release-recapture statistics was used to estimate the total number of OTUs within each sample. For principal coordinate analysis (PCA), all 16S rRNA gene sequences were imported into the ARB software package and aligned into a phylogenetic tree by Neighbour Joining which was used to perform clustering analysis using online UniFrac without abundance weighting [15], [16]. A p-test was performed in UniFrac to determine whether each sample was significantly different from others. All sequences will be deposited in the GenBank nucleotide sequence databases under the accession numbers JN609650 - JN612805. Student's t-test was used to test the significance of differences between groups or samples. Statistical significance was set at p<0.05.

## Results

In the first phase of the study, there was clustering of each individual's microbial communities in profiles where the 16S rRNA gene was digested with *Msp*I and *Rsa*I. There was no clustering in the profiles where *Hha*I was used. *Msp*I digestion provided the clearest clustering of individual's microbial communities and these profiles were used for further analyses.

**Figure 2:**
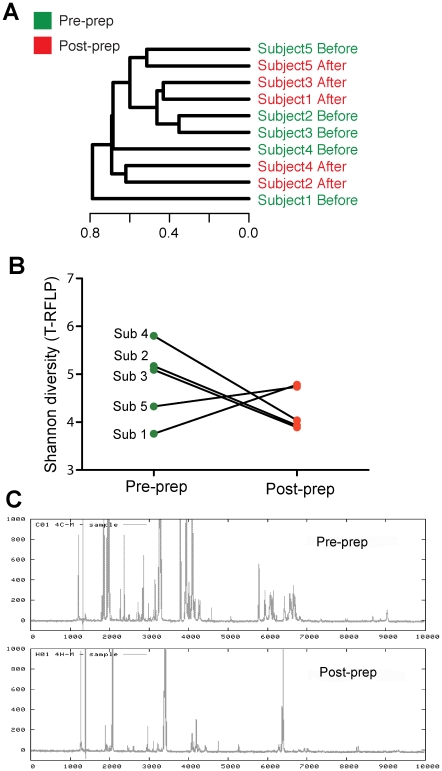
T-RFLP analysis demonstrated a change in the mucosa-associated microbiota after bowel preparation. (A) Dendrogram based on similarities between bacterial communities before and after bowel purge with Golytely preparation in all 5 study subjects within 2 weeks. Pre-prep communities are represented in green and post-prep communities are represented in red. Samples collected from the same subject are not clustered together. (B) Shannon diversity based on T-RFLP analysis was calculated. Decreased diversity was detected in three of five subjects. (C) Representative T-RFLP tracings from subject 2 before and after bowel preparation. The post-prep tracing shows an overall reduction in the diversity of the gut microbiota in this subject. (Sub represents subject.).

A dendrogram comparing the microbial communities in biopsies obtained from phase 1 subjects before and after bowel preparation is shown in Figure 2A. In four out of five subjects, microbial communities from biopsies collected before bowel preparation separated from communities from biopsies taken after bowel preparation. To further quantify the differences of bacterial populations between samples, the number of distinct terminal restriction fragments of the bacterial communities was used to calculate the Shannon diversity index. In three of the five individuals (subjects 2, 3 and 4), there was a marked reduction in the diversity of the T-RFLP tracings in the post-prep samples compared to the pre-prep samples (Figure 2B). In contrast, diversity was increased in two of the individuals after bowel preparation. The T-RFLP results therefore did not demonstrate a significant reduction in diversity after bowel preparation (p = 0.17). A representative dendrogram of T-RFLP tracings from subject 2 is shown in Figure 2C.

**Figure 3:**
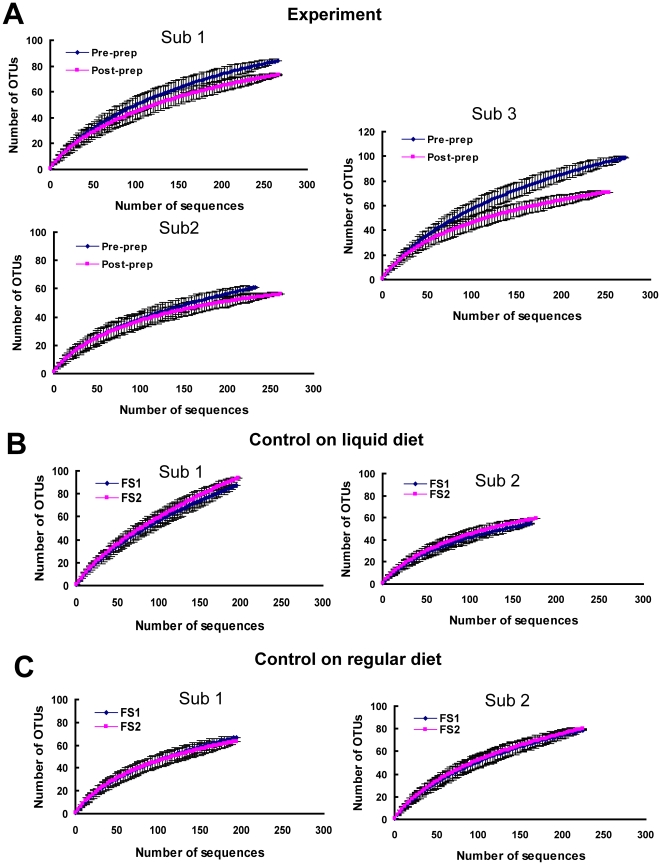
Rarefaction curve of phylotype richness. All subjects underwent two flexible sigmoidoscopies within one week. Each curve demonstrates the observed richness as additional clones are sampled and error bars represent 95% confidence intervals (CIs). Rarefaction curves from all three subjects in experimental group (A) show a decreased richness of bacterial phylotype in samples collected after bowel preparation compared to that in samples collected before bowel preparation (p<0.01, paired t-test). No difference of richness is found in the two control groups with two un-prepped flexible sigmoidoscopies (p>0.05, B and C). (Sub represents subject. FS1 represents the first flexible sigmoidoscopy and FS2 represents the second flexible sigmoidoscopy.).

In the second phase of the study, 16S rRNA gene clone library and sequencing analysis of the 14 samples obtained from the 7 subjects enrolled in the experimental and control groups. A total of 4,032 clones were randomly picked from 14 clone libraries and sequenced. After quality control and sequence assembly, 3158 clones yielded approximately 650 base pairs of partial 16S rRNA gene sequences that were used for analysis (Table S1). 16S rRNA gene sequences were assigned into operational taxonomic units (OTUs) or phylotypes at a similarity cutoff value of 97% using the DOTUR program. Rarefaction curves were used to compare the observed richness of OTUs between samples collected from the first and second flexible sigmoidoscopies in both control and experimental groups. In the experimental group, there was a separation between the two rarefaction curves. In all three subjects in the experimental group, the number of observed OTUs was decreased in biopsies collected after colonic preparation compared to biopsies obtained before colonic preparation. Separation of rarefaction curves was not observed in the two control groups (Figure 3).

Because we observed a reduction in diversity after bowel preparation in all three subjects in our experimental group, we used a one-tailed t-test to measure this effect. The Shannon diversity index was significantly lower in the samples collected after bowel preparation compared to those collected before preparation (p = 0.04). There was not a significant decrease in the Shannon diversity index in samples collected during the second flexible sigmoidoscopy compared to those collected during the first flexible sigmoidoscopy in either control group (p>0.05 in both control groups, Figure 3).

**Figure 4:**
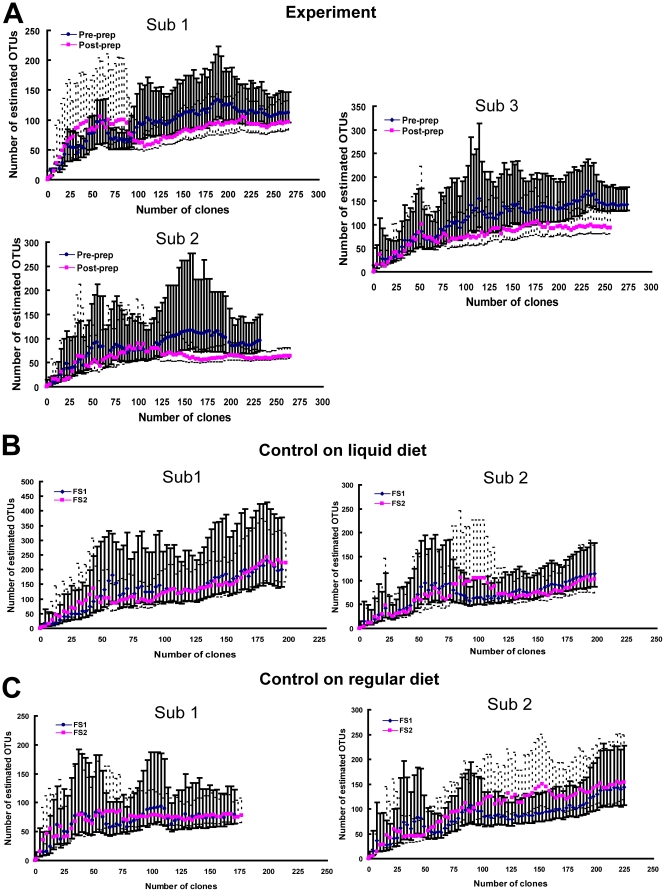
Chao1 estimates the total richness of bacterial community. (A) Total richness is decreased in samples collected after bowel preparation compared to samples collected without bowel preparation (p<0.01, paired t-test). This trend is not observed in the two control groups (B and C). Error bars represent 95% confidence intervals (CIs), which are calculated with the variance formula derived by Chao. (Sub represents subject. FS1 represents the first flexible sigmoidoscopy and FS2 represents the second flexible sigmoidoscopy.).

As the observed richness values from rarefaction do not necessarily correspond to relative total richness of community, the Chao1 richness estimator (which minimally estimates the total richness of a community) was used to assess and compare the total diversity of mucosa-associated microbiota between samples. As shown in Figure 4, Chao1 estimate curves from all samples leveled off at the end of OTU collection, suggesting that the end point value of Chao1 is a reasonable estimate of total richness. Although the confidence intervals did overlap at some points, as seen in Figure 4, the overall difference in the estimated richness in biopsy samples collected from the prepped colon compared to the un-prepped colon was significant using paired t-test (p<0.01). There were no significant differences in the Chao1 richness estimates obtained from the two sequential flexible sigmoidoscopies in either of the control groups (p>0.05, p>0.05)).

**Figure 5:**
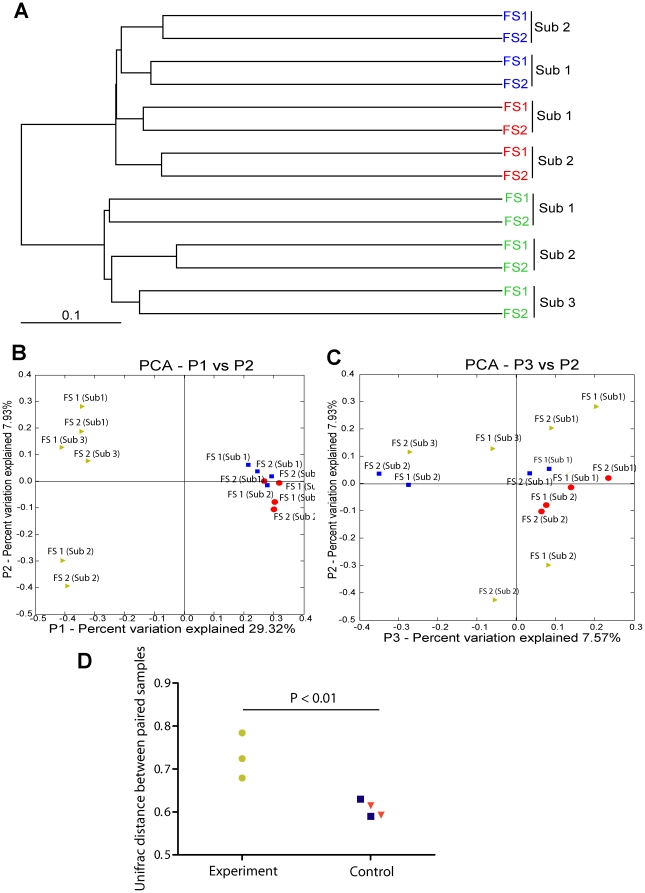
Unifrac analysis of colone libraries and sequencing. (A) Dendrograms based on the sequencing data. (B and C) Principal component analysis (PCA) of clone libraries. (B: P1 vs P2 and C: P2 vs P3) Each spot represents one sample in the PCA plot. The distance between samples represents the similarity between samples. The clustering of samples from two un-prepped flexible sigmoidoscopies (Subgroup 2 and 3) was slightly tighter than clustering of two samples from prep and un-prepped flexible sigmoidoscopies (Subgroup 1) in each subject. (D) The values of Unifrac distance between paired samples in experimental and control groups were compared. The distance between samples collected in the experimental group was significantly different. Yellow spots represent Subgroup 1 (experiment) in Phase II. Blue spots represent Subgroup 2 (control on liquid diet) in Phase II. Red spots represent Subgroup 3 (control on regular diet) in Phase II. (Sub represents subject. FS1 represents the first flexible sigmoidoscopy and FS2 represents the second flexible sigmoidoscopy.).

Because the richness analysis does not account for the similarity of phylogenetic composition between samples, we used Unifrac based clustering analysis to define the compositional difference between samples. A dendrogram was constructed from the sequencing data and showed that the microbiota from each individual consistently clustered together. This clustering of the microbiota in individuals was not seen in samples subjected to T-RFLP analysis, (Figure 5A). We believe the disparity between the two approaches is related to the lower sensitivity of T-RFLP. Thus, we put greater stock into the sequencing data. As shown in the principal coordinate analysis (Figure 5B and 5C), distances between coordinates represent the relative microbial structure similarity between samples. Paired samples collected from the experimental group had a relative large distance between each other compared to paired samples from the two control groups, (p = 0.0045, Figure 5D). The Unifrac p-test demonstrated significant phylogenetic compositional differences between pre- and post-prep samples in the experimental subjects. The differences between samples collected from the first and second flexible sigmoidoscopies in both control groups were not significantly different (Table 1).

**Table 1:** Comparison results of paired samples by p-Test in UniFrac.

	2FS 1(Sub 1)	2FS 2(Sub 1)	3FS 1(Sub 1)	3FS 2(Sub 1)	2FS 1(Sub 2)	2FS 2(Sub 2)	3FS 1(Sub 2)	3FS 2(Sub 2)	1FS 1(Sub 1)	1FS 1(Sub 2)	1FS 1(Sub 3)	1FS 2(Sub 1)	1FS 2(Sub 2)	1FS 2(Sub 3)
**2FS 1** **(Sub 1)**	-	0.52	0.00	0.00	0.00	0.00	0.00	0.00	0.00	0.00	0.00	0.00	0.00	0.00
**2FS 2** **(Sub 1)**	-	-	0.00	0.00	0.00	0.00	0.00	0.00	0.00	0.00	0.00	0.00	0.00	0.00
**3FS 1** **(Sub 1)**	-	-	-	0.15	0.00	0.00	0.00	0.00	0.00	0.00	0.00	0.00	0.00	0.00
**3FS 2** **(Sub 1)**	-	-	-	-	0.00	0.00	0.00	0.00	0.00	0.00	0.00	0.00	0.00	0.00
**2FS 1** **(Sub 2)**	-	-	-	-	-	0.96	0.00	0.00	0.00	0.00	0.00	0.00	0.00	0.00
**2FS 2** **(Sub 2)**	-	-	-	-	-	-	0.00	0.00	0.00	0.00	0.00	0.00	0.00	0.00
**3FS 1** **(Sub 2)**	-	-	-	-	-	-	-	0.55	0.00	0.00	0.00	0.00	0.00	0.00
**3FS 2** **(Sub 2)**	-	-	-	-	-	-	-	-	0.00	0.00	0.00	0.00	0.00	0.00
**1FS 1** **(Sub 1)**	-	-	-	-	-	-	-	-	-	0.00	0.00	0.00	0.00	0.00
**1FS 1** **(Sub 2)**	-	-	-	-	-	-	-	-	-	-	0.00	0.00	0.00	0.00
**1FS 1** **(Sub 3)**	-	-	-	-	-	-	-	-	-	-	-	0.00	0.00	0.00
**1FS 2** **(Sub 1)**	-	-	-	-	-	-	-	-	-	-	-	-	0.00	0.00
**1FS 2** **(Sub 2)**	-	-	-	-	-	-	-	-	-	-	-	-	-	0.00
**1FS 2** **(Sub 3)**	-	-	-	-	-	-	-	-	-	-	-	-	-	-

(1FS represents Subgroup 1 in Phase II. 2FS represents Subgroup 2 in Phase II. 3FS represents Subgroup 3 in Phase II. Sub is abbreviation for Subject.).

**Figure 6:**
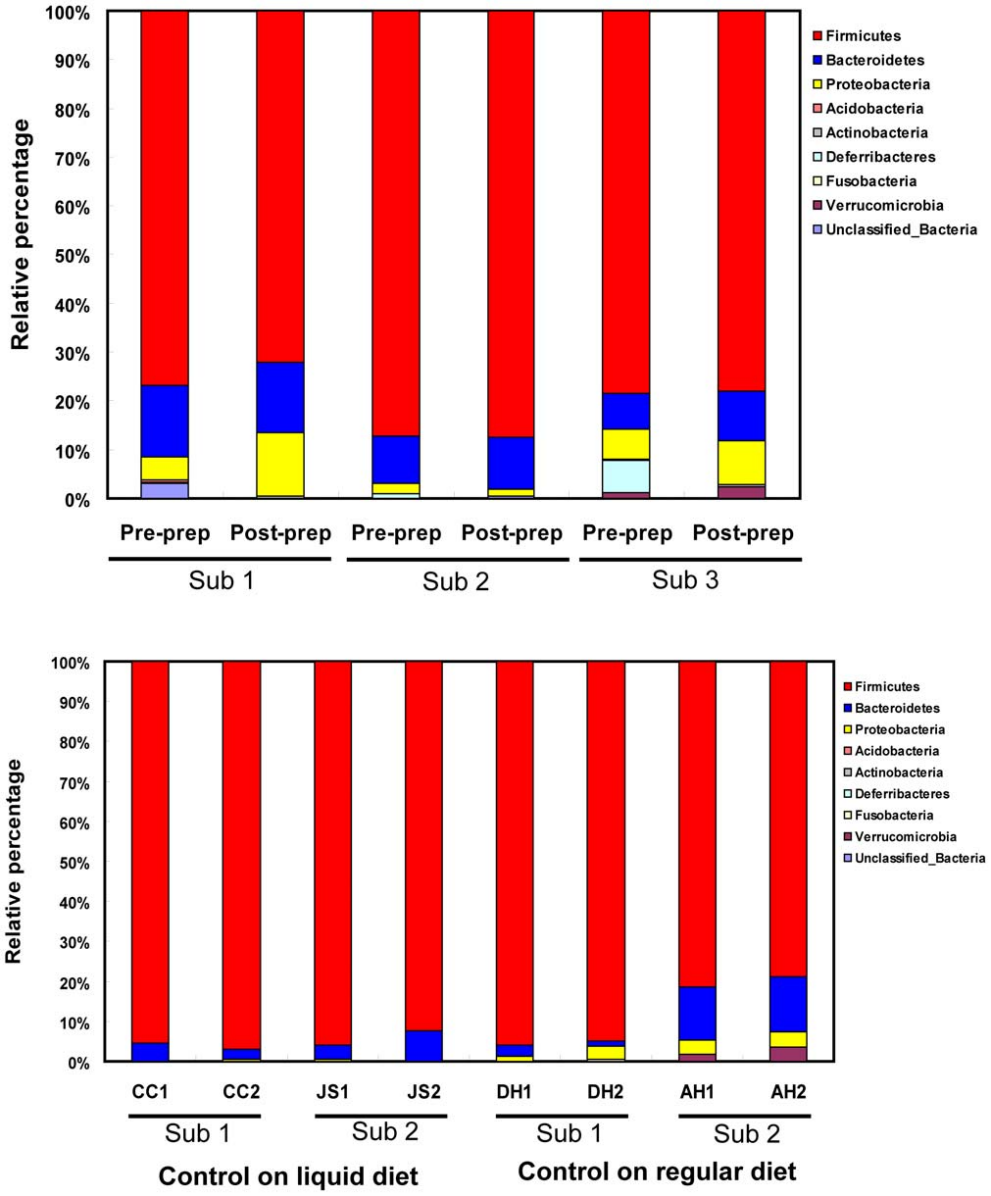
16S rRNA gene clone library sequence analysis of microbial communities in samples. Relative bacterial composition in mucosal sample from all three groups is shown at the phylum level. 16S rRNA gene sequences are grouped into different phyla using the RDP classifier tool at a default confidence threshold. (Sub represents subject. FS1 represents the first flexible sigmoidoscopy and FS2 represents the second flexible sigmoidoscopy.).

The taxonomic outlines of each sample were also examined. The majority of organisms in all samples were classified into two phyla Firmicutes and Bacteroidetes, which is consistent with other studies using biopsies from prepped human colon. Organisms from the phyla Proteobacteria, Verrucomicrobia, Fusobacteria, Actinobacteria, Acidobacteria, and Deferribacteres were minor populations in these samples. As shown in Figure 6, bowel preparation did not significantly change the phylogenetic structure at the phylum level. However, at the genus level, changes were more prominent between samples from prepped and un-prepped colons but not in the control samples (p<0.05 in the experimental group using classification tool in RDP, Figure S1). Despite significant variations of mucosa-associated microbiota among individuals, we did not find consistent changes in microbiota as a result of bowel preparation. For example, *Blautia* was decreased by bowel preparation in subjects 1 and 2, but not changed in subject 3. *Butyricicoccus* was decreased in subjects 1 and 3, but not detected in subject 2. However, *Mucispirillum*, a group of bacteria present in low abundance in the un-prepped colon, consistently disappeared completely after bowel preparation in all three subjects.

## Discussion

Significant advances have been made in technology and bioinformatic analysis allowing investigators to study and better understand complex microbiota. Despite these advances, many challenges remain. Among these challenges, include obtaining undistorted and representative samples from the human gut. While many researchers have suspected that colonic lavage distorts the enteric microbiota, there are many challenges in obtaining samples from an un-prepped colon. Our study demonstrates that colonic lavage may distort both the diversity and structure of the enteric microbiota and these findings question the reliability of sampling by luminal fluid aspiration and mucosal biopsy after colonic lavage.

We observed a reduction in overall diversity after colonic lavage in the mucosal samples obtained from three of the five subjects in the first phase of our study. Microbial diversity appeared to increase after colonic lavage in the other two subjects. This observed increase in diversity in the latter two subjects likely accounts for the similar Shannon diversity scores in the prepped and un-prepped samples. These initial observations prompted us to repeat our study and include control groups to determine if there are normal fluctuations in an individual's colonic microbiota and to determine if a clear liquid diet alone alters the enteric microbiota. In the second phase of our study, we shortened the time interval between flexible sigmoidoscopies to minimize any potential fluctuations in an individual's colonic microbiota over time. In order to achieve greater resolution than that achieved with T-RFLP analysis, 16S rRNA gene clone library and sequencing analysis were performed on samples collected from subjects enrolled in the second phase of the study. There was a decrease in richness and microbial structure similarity after colonic lavage. These changes were not observed in the two control groups, confirming that the trend towards a decrease in microbial diversity observed in patients taking a bowel prep is not due to clear liquid diet alone or normal fluctuations in the colonic microbiota.

Although our sample size is small, it appears that colonic lavage does affect the enteric microbiota, albeit this effect is not consistent among individuals. The variation in response to colonic lavage observed in our study may be attributable to large differences between individual's enteric microbiota, effectiveness and completeness of colonic lavage, and inter-individual differences in factors that may influence the enteric microbiota such as diet, medications, and lifestyle.

Previous studies of the human microbiota demonstrated a modest diversity of colonic microorganisms relative to other microbial communities, 62% of which appeared to be unique based on 16S rRNA gene sequence [17]. In the study by Eckburg and colleagues, the fecal microbiota was significantly different from the mucosa-associated microbiota in all three subjects [7]. The differences among mucosal samples from proximal to distal colon were not readily apparent, although a few subtle differences were noted. Overall diversity appeared to be similar. These results are somewhat surprising in light of the differences in properties and function of the proximal and distal colon [10], [18]. These regional differences might be expected to define “assembly rules” that would participate in selection and niche stability of certain microbial species [10]. Our group characterized the structure of the colonic mucosal-associated microbiota and the metagenomic profiles of the microbiota in the various regions of the colon in a healthy individual who underwent an un-prepped colonoscopy. We noted significant regional differences of the colonic microbiota [18]. We also noted axial variation of the microbiota; the microbial communities adherent to the mucosal surface differed from those in the lumen of the colon. Two recently published mouse studies have described differences in the community structure and diversity of the mucosal-associated microbiota present in the proximal murine colon compared to the distal murine colon [10], [18]. Based on the observations of these recent studies, we believe that the findings of Eckburg et al. may be the result of dilutional skewing and reduction of diversity introduced by colonic lavage. The vigorous actions of the lavage solution appear not only to affect overlying mucosa-associated microbes, but might also affect the integrity and abundance of overlying mucus of the colonic epithelium. Histological analysis of the colons of mice lavaged with polyethylene glycol solution have shown a dramatic loss of mucosal-associated microbes and a destruction of the associated biofilm, (L. Lichtenstein, Y. Wang, E. B. Chang, unpublished observation. Figure S2). Alteration of mucus can dramatically affect the diversity and structure of associated microbial ecosystems that depend on it for attachment, stability, and nutrient source. It should however be noted that the mucosa-associated communities are not as distinct in the human distal colon compared to the mouse colon.

Our study demonstrates a variable treatment effect of colonic lavage on the mucosa-associated enteric microbiota independent of the clear liquid diet or normal variation of the enteric microbiota with time. Based on our findings, we believe that future studies of the enteric microbiota should take into consideration the confounding effects of colonic lavage. Studies that had previously acquired microbiota samples under these circumstances may have to be revisited under less perturbing conditions. We recommend that future investigations of the human enteric microbiota include un-prepped subjects in whom the natural state of the colonic microbiota can be preserved and observed. Acquisition of left sided colon samples should not be a major problem, but obtaining right side colonic samples will be more technically demanding; potentially requiring conscious sedation and increasing risk and duration of colonoscopic procedures. Nonetheless, in the hands of an experienced endoscopist, full colonoscopy in un-prepped individuals is feasible. Our group has an 80% success rate reaching the cecum in un-prepped patients.

In summary, we report that the routine practice of colonic lavage may significantly alter the mucosa-associated microbiota of the distal human colon. While the effects are obvious in some individuals, the effects of colonic lavage can be unpredictable. Given that colonic lavage has the potential to distort the enteric microbiota, we recommend that future studies of the human enteric microbiota be performed on the un-prepped colon where the natural state of both luminal and mucosa-associated microbiota is most likely to be retained.

## Supporting Information

Figure S1 Relative abundance of bacterial genus in all samples. (Sub represents subject. FS1 represents the first flexible sigmoidoscopy and FS2 represents the second flexible sigmoidoscopy.)
(TIF)

Figure S2 Effect of polyethylene glycol prep on murine colon. Following purge with polyethylene glycol prep, mice had dramatic loss of mucosal-associated microbes and destruction of biofilm. Depletion of goblet cells was also noted in post-prep sample. These changes were not seen in the mice lavaged with normal saline.
(JPG)

Table S1 (1FS represents Subgroup 1 in Phase II. 2FS represents Subgroup 2 in Phase II. 3FS represents Subgroup 3 in Phase II. Sub is abbreviation for Subject.)
(DOC)
