# Supplementary figures and images for: Standard Colonic Lavage Alters the Natural State of Mucosal-Associated Microbiota in the Human Colon
Source: PLoS One. 2012 Feb 28;7(2):e32545. doi: 10.1371/journal.pone.0032545 (PMC3289660; doi:10.1371/journal.pone.0032545)

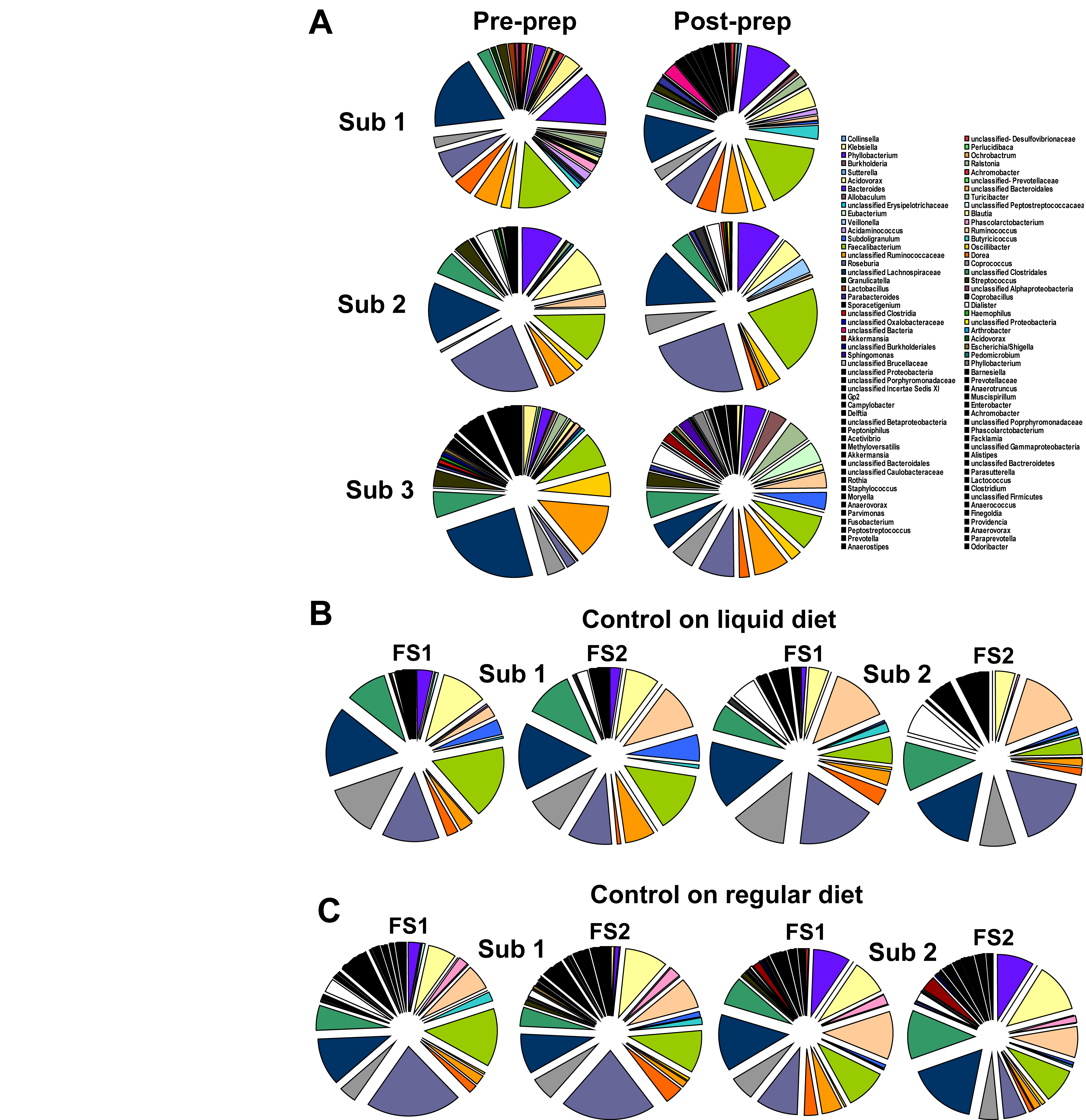

Supplement: Figure S1 — Relative abundance of bacterial genus in all samples. (Sub represents subject. FS1 represents the first flexible sigmoidoscopy and FS2 represents the second flexible sigmoidoscopy.) (TIF) [file pone.0032545.s001.tif]

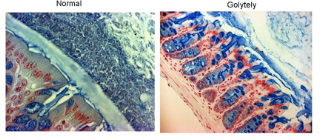

Supplement: Figure S2 — Effect of polyethylene glycol prep on murine colon. Following purge with polyethylene glycol prep, mice had dramatic loss of mucosal-associated microbes and destruction of biofilm. Depletion of goblet cells was also noted in post-prep sample. These changes were not seen in the mice lavaged with normal saline. (JPG) [file pone.0032545.s002.jpg]
