# Supplementary material for: Standard Colonic Lavage Alters the Natural State of Mucosal-Associated Microbiota in the Human Colon
Source: PLoS One. 2012 Feb 28;7(2):e32545. doi: 10.1371/journal.pone.0032545 (PMC3289660; doi:10.1371/journal.pone.0032545)
Supplement: Table S1 — (1FS represents Subgroup 1 in Phase II. 2FS represents Subgroup 2 in Phase II. 3FS represents Subgroup 3 in Phase II. Sub is abbreviation for Subject.) (DOC) [file pone.0032545.s003.doc]

**Table S1** Summary of results from 16S rRNA gene clone library

| Sample name | Number of sequences | Number of OTUs | Number of Chao1 estimated OTUs | Shannon diversity |
| --- | --- | --- | --- | --- |
| **1FS 1**  **(Sub 1)** | 268 | 84 | 112 | 3.85 |
| **1FS 2**  **(Sub 1)** | 269 | 73 | 97 | 3.62 |
| **1FS 1**  **(Sub 2)** | 234 | 61 | 98 | 3.43 |
| **1FS 2**  **(Sub 2)** | 264 | 56 | 64 | 3.31 |
| **1FS 1**  **(Sub 3)** | 273 | 99 | 142 | 4.17 |
| **1FS 2**  **(Sub 3)** | 255 | 71 | 93 | 3.81 |
| **2FS 1**  **(Sub 1)** | 196 | 87 | 201 | 4.03 |
| **2FS 2**  **(Sub 1)** | 199 | 94 | 224 | 4.11 |
| **2FS 1**  **(Sub 2)** | 197 | 67 | 113 | 3.73 |
| **2FS 2**  **(Sub 2)** | 198 | 63 | 104 | 3.48 |
| **3FS 1**  **(Sub 1)** | 174 | 55 | 79 | 3.48 |
| **3FS 2**  **(Sub 1)** | 178 | 59 | 77 | 3.59 |
| **3FS 1**  **(Sub 2)** | 227 | 79 | 144 | 3.91 |
| **3FS 2**  **(Sub 2)** | 226 | 80 | 154 | 3.98 |
